# Gene regulatory networks: from correlative models to causal explanations

**DOI:** 10.1038/s41576-026-00939-1

**Published:** 2026-03-09

**Authors:** Rory J. Maizels, James Briscoe

**Affiliations:** The Francis Crick Institute, 1 Midland Road, London NW1 1AT, UK

## Abstract

Gene regulatory networks (GRNs) explain how the genome controls cellular behaviour and tissue morphogenesis, serving to connect molecular mechanism to functional output. Single cell technologies now provide descriptions of these networks with unprecedented detail, but this is creating a dilemma for the field: we are discovering gene regulatory systems that are too complex for our existing conceptual frameworks. GRNs, which should provide mechanistic explanations, are increasingly reduced to statistical correlations - ‘hairballs’ that fail to capture molecular causation. Here, we explore why this dilemma exists and propose a path forward. We argue that methods in *representation learning* can be used to model GRNs, without needing to capture every molecular detail. For this framework, we advocate three linked principles: (1) models must be inherently mechanistic with structures grounded in cellular, evolutionary and experimental biology; (2) molecular principles and constraints must be used to reduce the solution space for learning GRN models; and (3) more sophisticated forms of experimental perturbation and synthetic biological engineering are needed to train models and test predictions. By reimagining GRNs through these principles, we can bridge the gap from data abundance to new conceptual understanding.

*“No observations on single genes can ever illuminate the overall mechanisms of development of the body plan or of body parts except at the minute and always partial, if not wholly illusory, level of the worm’s eye view.”*

– Eric H. Davidson

## Introduction

With their systematic mutational screen of segmentation in Drosophila embryos, Nusslein-Volhard and Wieschaus provided a catalysing insight into the molecular and genetic basis of development^1–3^. Their work revealed that developmental processes such as body segmentation can be orchestrated by a surprisingly small number of genes; underneath the complexity of the developing organism lies elegant simplicity. Later work showed that developmental genes are highly conserved across species, and that changes in their regulation are responsible for the evolutionary divergence of body plans^4–6^. These discoveries laid the foundations for understanding how body plans evolved and how they are encoded in the genome.

In the following decades, we have built a deeper and more detailed understanding of the developmental genetics of tissue formation, leading as far as a call for a Perturbation Cell and Tissue Atlas^7^ that will extend the genetic screen to its logical conclusion of documenting the role of every gene in every tissue.

Yet in these decades, a gap has opened up between the reductionist approach of studying genetic function gene-by-gene and a mechanistic understanding of the developmental logic that these genes control. This is because genes are not fixed entities with singular roles; they cannot be considered independent units of causality^8–10^. Function is not an inherent property of a gene but a context-dependent property of the system in which it is expressed. No gene is an island: all are interdependent, interacting and communicating in complex networks^11^. It is through the dynamics of these networks, rather than through any single gene, that developmental form emerges.

This view is not new. Over the past half-century, the concept of the gene regulatory network (GRN) has become central to developmental biology^9,12,13^. Broadly, a GRN is a system of molecular genetic regulators that act in concert to drive particular cellular outcomes. The basic form of a GRN is a set of transcription factors (TFs) that act at the cis-regulatory elements (CREs) of other genes (although this definition can be generalised to accommodate protein modifiers, signalling pathways, the three-dimensional organisation of chromatin^14^ and so forth). Fundamentally, the GRN concept promises a systems-level perspective on how genes and their regulators govern cellular functions and tissue morphogenesis. It offers a holistic description of regulatory genes operating together (see Box 1). These holistic descriptions can explain behaviours that could not be produced by a single gene, such as oscillations^15–17^, stripes^18–22^ and switch-like responses^23–25^.

GRNs are also a crucial evolutionary idea, with fundamental principles of GRN organisation arising from comparisons between species^13,26–28^. It has been suggested that developmental GRNs contain evolutionarily ancient sets of core regulatory genes, known as ‘kernels’^13^ or ‘Character Identity Networks (ChINs)’^27,28^ that drive tissue specific developmental programmes. Thus, although the components of a GRN can diverge between species, the systemic logic of the GRN system can remain conserved, so that the GRN acts as the molecular and mechanistic basis of homology^29^. The human hand and the bird’s wing have distinct morphologies and functions; it is their homologous GRN kernels that reflects their shared evolutionary origin. These evolutionary perspectives demonstrate the importance of a systems-level perspective: GRNs are more than the sum of the molecular relationships they contain. They provide mechanistic control of biological function. They act as a dynamic map from genotype to phenotype^8^. Building an understanding of these systems that can explain this mechanistic control, this dynamic map of a GRN will be just as important as curating the sets of interactions contained within it.

## Causal versus correlative GRNs

The original formulation of the GRN concept was inherently causal, constructed through experimental interventions on the genome. By specifying how the activity of one component changes under specific perturbation of another, causal relationships were established at a molecular level^30^. Doing this iteratively builds up a set of relationships that collectively create a ‘gene regulatory network.’ In this way, the GRN concept explains how complex cell- and tissue-level phenotypes emerge from molecular processes. It provides a description of how genes behave collectively; a theoretical framework for viewing the cell not just as a bag of genes but a complex, finely tuned control system.

The goal of building a causal understanding of GRNs goes beyond identifying the causal relationships between regulatory components, how the presence of one molecule leads to production of another, and more broadly asks: how does the gene regulatory system *cause* the cell phenotype? The field of causal learning provides a tool box for defining and analysing interactions in data with causal semantics. But in this instance, we must also consider what interventions on gene regulation will be required, what data must be collected, and how must we represent GRNs for this emergent level of collective causality to become evident.

With the collective behaviour of genes in mind, the advent of genomics technologies that measured expression across thousands of genes simultaneously created considerable excitement in the field of gene regulation modelling^31,32^. Yet these methods have also highlighted the magnitude of the challenge: genome-wide measurements demonstrate how large developmental GRNs can be, with dozens of transcription factors^33,34^ interacting across thousands of binding sites^35,36^.

Single-cell resolution has revealed the true extent of dynamism, stochasticity and heterogeneity in the developing embryo^37–40^.

In light of these findings, the traditional approach of systematic intervention seems infeasible. This has led to the rise of computational GRN models^41–44^. With these ‘statistical GRN’ models, a common thread is that the number of parameters being learnt (the number of possible interactions) is greater than the number of interventionalist observations (for example, the number of genetic knockouts). As such, these models are built to infer relationships from statistical patterns in the data, such as co-variance between genes across cells or samples. They can successfully identify important factors in the network, but fall short of the desired explanatory power of mapping from genotype to phenotype.

This situation presents a problem to the field. For GRN models to provide more than just a list of molecular interactions, we need systems-level understanding that is mechanistic and rooted in the methods of causal discovery. But building such models for more than a handful of genes is extremely challenging. On the other hand, statistical GRNs trained on genomics data can handle the complexity of modern datasets, but do not provide any systems-level understanding of how GRNs control cellular decisions.

Here, we urge a return to the view of GRNs as mechanistic explanations. We acknowledge that ‘big data’ methods such as single-cell genomics will be needed to capture the full complexity of gene regulation; but we require new approaches to distil the complex, high-dimensional picture of these datasets into interpretable mechanisms and clear explanations.

Machine learning methods will be crucial for this effort. In particular, causal representation learning^45^ techniques can decompose the complexities of large datasets into key variables for the question at hand. Experimental developments offer exciting opportunities, too: synthetic biology offers the chance to understand through engineering, building synthetic networks and cis-regulatory perturbations that expand the space of systems that we can explore. Through these combined efforts we can create new mechanistic models of gene regulation that span the scales from DNA sequence to cellular phenotype and evolutionary dynamics.

## Modelling gene regulatory networks

**Figure 1:**
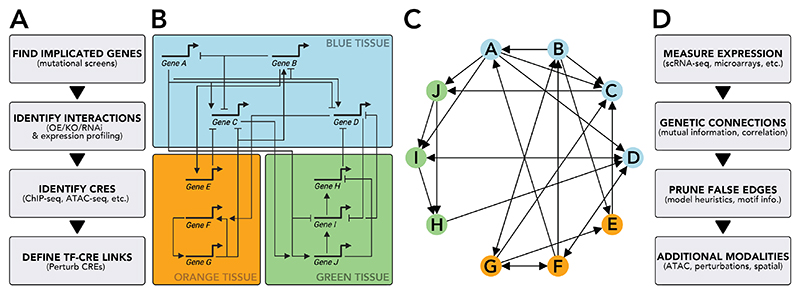
Classical versus data-driven GRN construction. A. A classical pipeline to construct a mechanistic GRN model. The workflow is often highly iterative and experimental, with relevant genes first identified and the relationship between genes experimentally tested. B. An example of a mechanistic GRN model: gene regulation through inductive or repressive interactions (arrows and bars, respectively); genes are functionally organised into modules which are responsible for controlling biological phenotypes. C. The same network represented as a statistical GRN, with interactions represented as arrows. D. A workflow for statistical GRN inference: data is collected and putative networks are constructed from statistical relationships between genes. Edges that are deemed to be indirect are pruned, and additional modalities of data can be incorporated either in training or validation.

Conventionally, the construction of GRN models is an iterative process of systematically identifying regulatory genes and elements, charting out expression patterns and establishing regulatory interactions through genetic perturbation^46–48^ (Figure 1A). The advent of high-throughput sequencing technologies led to GRN inference algorithms that expedited this process (Figure 1C) by capturing correlative patterns between genes across many samples (or many single cells), circumventing the need for piecewise reconstruction of regulatory connections (Figure 1D). Early methods, such as GENIE3^50^ and MRNET^51^, were designed for use with microarray or bulk RNA sequencing data, and were benchmarked using synthetic datasets or simple bacterial datasets where a true network structure was known. The performance of these methods was quantified through comparison to these ground truths, with limited application to real biological questions. With single-cell RNA sequencing came a host of new methods, applying techniques from Bayesian modelling^52^ to dynamical systems theory^53^, sometimes integrating pseudo-temporal^54^ or RNA velocity^55^ information (see Glossary Box3). These methods saw greater biological application, for example identifying novel genes that appeared to be involved in disease states. However, independent benchmarking studies of GRN inference tools revealed widespread poor performance^56^, with methods often performing no better than simple baselines or random guesswork^57^. Indeed, a recent study has demonstrated that, even with single-cell resolution, gene expression data alone is insufficient to control for false discoveries in GRN inference^58^.

The advent of multi-omics approaches that simultaneously measure gene expression and chromatin accessibility promised exciting improvements^41^. Methods such as SCENIC+^44^, Dictys^43^ and CellOracle^42^ use multi-modal single cell data to model GRNs, and have been used to identify key transcription factors and important enhancer regions. In some cases, they have been used to predict the effect of perturbing well-studied differentiation driver genes or to find new important cell fate transcription factors^42^. Yet, despite the promise of multimodality, these data bring more issues for modelling. Inferring GRNs from chromatin accessibility requires two additional inference tasks for each chromatin region: inferring which TF binds to it and which downstream gene it regulates. Predicting TF binding from sequence alone is not straight-forward^59,60^, as binding motifs can be highly degenerate (one TF can bind a range of sequences, one motif can be bound by many TFs^61^). An enhancer can be tens to hundreds of kilobases away from the gene that it regulates^62^. Perhaps as a result, recent independent benchmarking revealed that multimodal GRN inference methods have limited robustness, high sensitivity to user-supplied parameters, and performed poorly at perturbation-based causal predictions^63^.

## The challenges of modelling GRNs

The basic formulation of a GRN model is a network graph, where each node is a gene, and each edge is an interaction between two genes. The challenge of GRN inference is clear from the nature of these graph structures: if interactions are directional and self-interactions included, the number of possible interactions in a network of *n* genes is *n*^2^, and the number of possible network topologies is $2^{n^{2}}$. So, a ten-gene network has 100 possible interactions and more than 10^30^ possible topologies. Systematically deleting each gene in the network generates only 11 observational conditions (10 knockouts and wild-type) but 100 interaction parameters need to be learnt. And if, after experimentally testing each of these 100 interactions, one is 95% confident of each interaction estimation, this still only gives 0.6% confidence in overall network structure. Making matters worse is the fact that GRNs are dynamic processes with time-dependent interactions and feedback loops^64^, meaning any static representation of a network (for example, a conventional graph of nodes and edges) would not necessarily capture the behaviour of the network^65,66^.

**Figure 2:**
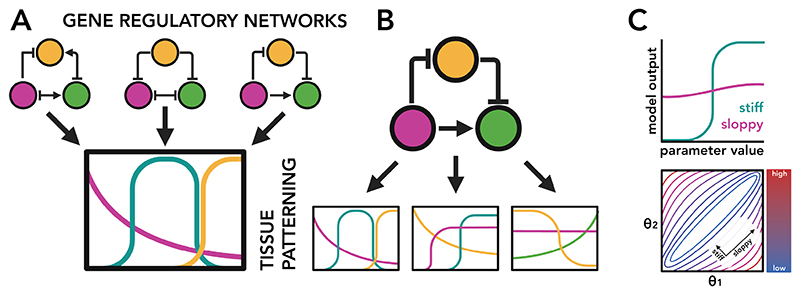
Challenges of GRN modelling A. Different GRN structures (three-node networks) can produce the same pattern of expression and tissue phenotype (represented as box of expression values through time/space). B. Conversely, the same GRN structure can produce different patterns depending on its parametrisations (strength of genegene interactions), contexts (boundary conditions) and initial conditions. C. The challenge of ‘sloppy models’. Top: In these cases, the model responds very sharply to changes in some parameters (stiff parameters) while hardly responding at all to others (sloppy parameters). Bottom: in parameter space, sloppy parameters can be visualised as directions in which the the model output does not change, the contour map is unchanging (in this example from bottom-left to top-right.

Theoretical work in systems biology and dynamical modelling has revealed a number of challenges faced when constructing models of complex systems (such as GRNs) that are only partially observed (as is the case with all biological datasets). Mathematical models can suffer from a phenomenon known as structural non-identifiability^67,68^, where different sets of model parameters generate the same output, making it impossible to determine the ‘correct’ parameter solution. A related but distinct^69^ phenomenon is that of ‘sloppy models’^70,71^, where certain parameters in a model can change by orders of magnitude without impacting the model’s output (Figure 2C).

These phenomena are related to a more general problem of ‘dynamical equivalence’^72,73^ where different models generate equivalent dynamics, making the task of identifying the correct model structure intractable. It has been shown that many different GRN structures are capable of generating the same patterning behaviour^74–76^ (Figure 2A). Similarly, slight parameter variations with a single GRN structure can create very different model behaviours^15,77^ (Figure 2B). One cannot expect a one-to-one mapping between the structure of genetic interactions within a GRN and the behaviour of the GRN as a whole. These problems worsen as the number of parameters in the model increases.

Even an accurate GRN graph model would not provide a complete, objective depiction of the reality of gene regulation. Many aspects are ignored in these models – from spatio-temporal dynamics to epigenetic regulation to transcription factor co-operativity, depending on the model. These aspects are deliberately ignored; they are abstracted with the assumption that they will be sufficiently captured by the parameters of the model. This abstraction is necessary: it makes the system tractable for analysis.

Understanding this act of simplification allows us to ask whether our chosen level of abstraction captures the biological phenomena we wish to study. The choice to abstract molecular details and represent GRNs at the level of genetic interactions is based on the assumption that genes are the fundamental units of causality in cellular systems^8^. But biological function can be ‘emergent,’ arising from the dynamics and global structure of the GRN system itself^78–81^. Oscillations, switch-like behaviours, and Turing patterns are examples of emergent properties that are not evident from individual genes.

There is nothing to say that the emergent properties of a GRN cannot be described by an explicit and detailed model of every genetic component of the network. But these explicit representations may not provide the most informative depiction of these complex systems^82,83^. Understanding every individual interaction may not provide a clear explanation of the GRN’s cellular function any more than a complete understanding of the structure of amino acids explains the folding of proteins. Indeed, given the evident challenges associated with constructing GRN models, it is worth considering whether abstracting away the details of genetic interactions would help us to learn more about the functions that GRNs perform in cells.

What molecular organisation might be captured in an ‘emergent property’ of a GRN? It could be as simple as a quantification of the *ratio* of activities between two genes, rather than of the genes’ activities themselves (for example, the erythroid-myeloid fate decision depends on the stoichiometric balance between GATA1 and PU.1^84^). Other examples of emergent properties/mechanisms could include: many co-expressed genes that are induced in concert to engender a particular phenotype (such as the pigmentation gene module in melanocytes^85^); sets of related or duplicated components (such as the different Gli proteins that transduce Sonic hedgehog signals^86^ or the different enhancers that act cooperatively in the *α*-globin super-enhancer^87^); components that are molecularly disparate (e.g. DNA sequence and proteins) but act collectively to drive a specific cell-level phenotype (such as the sharp, position-specific stripe of expression driven by the even-skipped stripe 2 system in drosophila^88^ or the interferon-*β* enhanceosome that integrates NF-*κ*B and IRF signals in viral infection^89^); or a sub-circuit, within a wider network of transcription factors, that is responsible for a specific phenotype (such as the four-gene network of Pax6, Olig2, Nkx2-2 and Irx3 that drives ventral spinal cord patterning^86^).

In each example, molecular components form a larger functional unit, such that we could model the behaviour of the functional unit and abstract molecular details away. This coarse-grained approach could provide more robust models, reveal new forms of biological mechanism. It would provide a multi-perspectival way to study multiscale biological systems, with different abstractions for different questions. The challenge is to find how we can do this in a flexible, generic way, such that a single modelling approach would be suitable for all examples given above.

## Representational solutions

The challenge of modelling across different levels of granularity has been addressed in various scientific contexts. For example, in chemistry, coarse-grained modelling is used to produce molecular simulations that abstract away atomic information^90^, replacing particles with ‘pseudo-particles’ that only retain details that are relevant at the molecular or macromolecular level^91^. Comparably, Alphafold2^92^ abstracts amino acid chains as ‘triangular gases’, retaining only the core geometrical information required for modelling global protein structure.

But coarse-graining analysis to focus on ‘emergent properties’ can do more than just remove extraneous details. In studying signal processing system, higher levels of analysis can reveal broader design principles and functional structure. In neuroscience, Marr’s *levels of analysis*^93,94^ proposes three levels at which information processing systems can be understood. Highest is the *computational* level, which describes what problem is being solved by the system; the goals, constraints, success criteria. Next is the *algorithmic/representational* level, which describes how the system achieves its goal; how it processes inputs, builds useful representations, and uses these representations to create outputs. Finally, the *implementational* level, which describes the physical realisation of the system. Applying these levels of analysis to a radio^95^, we might say that the computational level of a radio is to deliver an audio programme to a listener. The implementational level will involve antennae, electronics, speakers, buttons, a power supply, and so on. Linking these is the algorithmic/representational level, which might describe how the radio selects a particular frequency with bandpass filtering, reads this signal with frequency discrimination, then processes this signal into data to send to speakers. The computational level describes why one might use a radio, the implementational level describes what a radio is composed of, but understanding how a radio works requires an understanding of the representational level.

**Figure 3:**
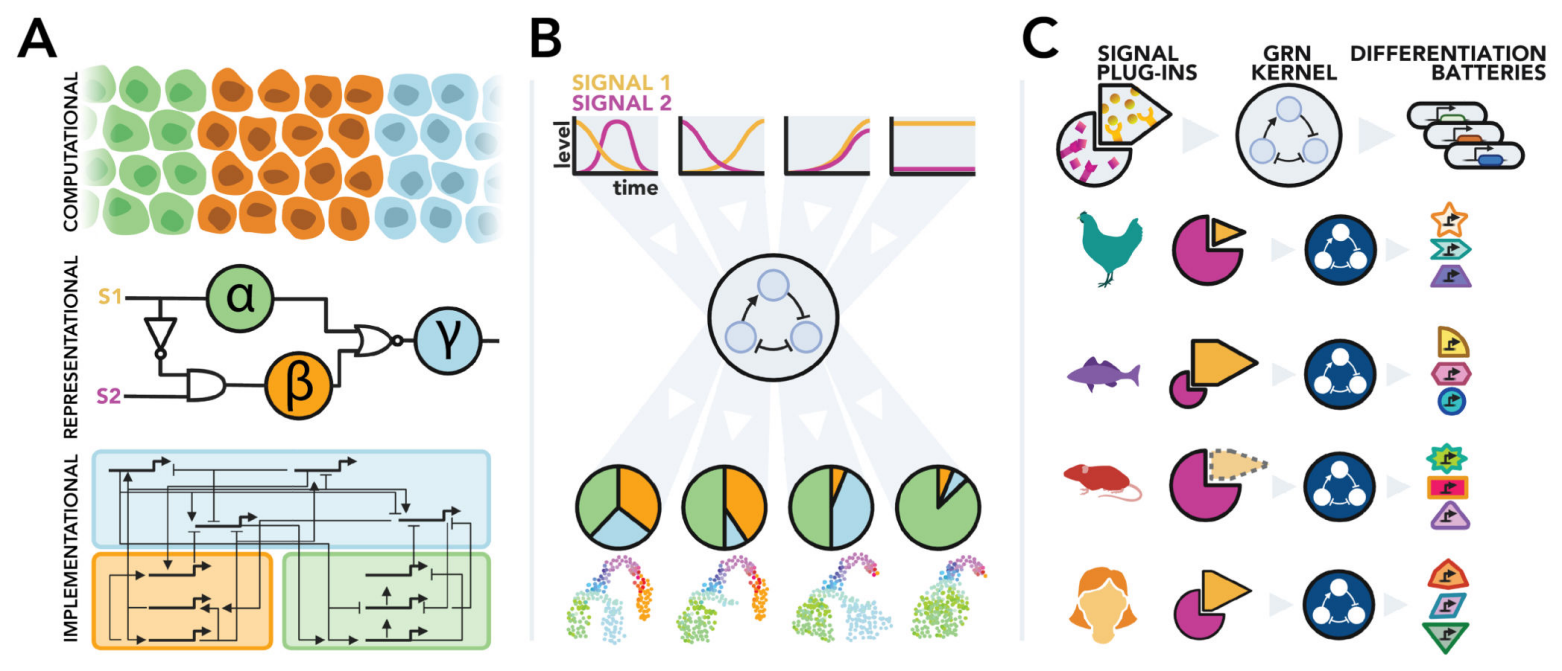
Representational descriptions of gene regulatory networks. A. Information processing description: Marr’s levels of analysis^93,94^ can be applied to the study of GRNs. An *implementational* level captures the physical realisation of the system, in the instance of GRNs, this is the explicit description of transcription factors and enhancers that mediate genetic interactions. Above this, a *representational* level describes the logic of how this physical realisation functions to interpret signals (S1 and S2) and achieve the system’s goals. This is visualised here as logic gate connecting abstract cell-type factors. Finally, a *computational* level describes the computational process being performed, in this instance the decoding of input signals to form a striped tissue pattern. B. Cellular signal interpretation descriptions: from a cellular perspective, GRNs can be thought of as processes that take signalling dynamics as input and then output cell type proportions. Constructing mechanistic models at the level of signal interpretation could describe the cellular function of GRNs without needing to explicitly model the underlying genetic interactions^102–104^. C. Evolutionary kernel description: GRNs consist of ‘plug-ins’ which are re-usable modules, such as signalling pathways that provide inputs; ‘kernels’ which contain the core functional logic of the GRN, and ‘differentiation batteries’ which are responsible for executing the downstream consequences of the GRN. The different functions of these modules are reflected in their evolutionary dynamics: kernels are highly conserved across species due to being functionally critical to tissue formation (visualised here as the unchanging blue network across species. Plug-ins display higher variability, particular in the contexts in which they are deployed across tissues in the organism (demonstrated here as the changing size and strength of the different modules). Differentiation batteries display the highest level of variability; they do not feed back into the GRN, and so are free to evolve and adapt to provide species-specific outputs. Capturing the evolutionary dynamics of GRN components could thus inform the functional role the components perform in the network.

This framework can similarly be applied to the signal processing performed by gene regulatory networks^96,97^. A cell-, tissue- or organism-level description of what processes a GRN controls; the phenotypes it drives, the contexts in which it functions. A molecular level that describes the proteins, cis-regulatory elements, and epigenetic components that construct the GRN; and connecting them, a representational level that describes how these different components organise, how input signals are mapped to output expression programs^96^, how this input-output mapping creates organismic function out of molecular components (Figure 3A).

To build this representational layer, understanding how molecular components are organised is crucial. In this respect, GRNS have been shown to possess structure and organisation^26,98^: task-specific sub-circuits provide a form of modularity^99,100^. These sub-circuits are organised in a hierarchical fashion that reflects the evolutionary and functional structure of the GRN^101^, while the sequential progression of metastable cell-states through development creates another form of hierarchy between cell-state specific sub-circuits. That hierarchy and modularity exist in this functional way, connecting to how GRNs operate to drive cellular decisions, suggests that GRN architectures are reducible and decomposable. Grouping genes into modules and structuring cell states into hierarchies naturally provides a bridge from genetic to cellular scales of function.

Next comes the question of how the system behaves. At the molecular level, behaviour is just the dynamics of components through time, perhaps extending to include interactions between components. But a more systemic, representational idea of GRN behaviour must map the system’s inputs to outputs.

What is the simplest model that can recapitulate outputs based on inputs? And then; how might the activity of components link to this input-output map?

The third question (more relevant to GRNs than radios) is to ask how the system evolved. Throughout evolution, neutral or even mildly deleterious mutations can accrue^105,106^ and cis-regulatory sequences vary considerably between species^107,108^, but mutations that impact the GRN’s representational behaviour (and thus computational function) are more likely to produce a dead end. Many developmental GRNs are built around ancient, stable cores of transcription factors (‘kernels’^13^ or ‘ChINs’^27,28^) that control key decisions. Feeding into these kernels are signalling input modules (termed ‘plug-ins’ or ‘I/O switches’) which show greater variability across species, though are often repeated across different contexts within an organism^13^. And responding to kernel activity are ‘differentiation batteries;’ downstream effector genes that execute the network’s output without feeding back into the regulatory system^13^. These genes show the highest variability across species, as they are not constrained by downstream regulatory logic and can evolve to execute species-specific ‘character states’ of a tissue. Just as the evolutionary dynamics of residues in a protein can provide structural information^109–111^, the evolutionary dynamics of genetic components could describe their role within the wider functional context of the GRN^112^. Over evolutionary time, developmental systems drift through different network configurations to produce equivalent outputs, rewiring connections while maintaining overall function^113^. The constraints that guide this drift process, and the correlative patterns that are created by it, could provide valuable prior information for modelling the structure and function of gene regulatory networks in development^114–116^.

Taking a ‘representational’ approach to modelling gene regulatory systems would help to reduce the solution space for models. But the benefit could be more fundamental. The approach moves from asking ‘what is a GRN made of?’ or ‘what is the structure of a GRN?’ to instead asking ‘how does a GRN map inputs to outputs, and how does this achieve the cell’s broader function?’ In doing so, this approach can shift focus towards design principles, cellular function and evolutionary dynamics, connecting the study of GRN structure with fundamental questions of biological purpose and origin.

The parameters of such a representational model of gene regulation would not necessarily capture distinct molecular entities or properties, such as proteins or reaction kinetics. The challenge, then, is to find biological constraints that ensure these models can be trained in a robust, principled way.

## Abstract representations and dimensionality reduction

In fields such as single-cell genomics, it is already common practice to visualise biological systems with abstract representations. Dimensionality reduction approaches such as PCA, UMAP^117^ and t-SNE^118^ condense the thousands of variables and observations into a more digestible two-dimensional depiction. Dimension reduction is also a common step in machine learning pipelines for statistical tasks such as multi-modal data integration^119–122^, batch correction^123–125^ and perturbation prediction^126–129^.

While reliance on low-dimensional visualisations can introduce distortions into analysis^130^, the principle motivating these approaches is that the number of variables required to properly describe biological systems is considerably less than the number of features one can measure of it. In other words, biology exists in a lower dimensional space than the full dimension of observable features (known beyond biology as the *manifold hypothesis*^131^). Biological features are correlated and interdependent; the system is constrained to fewer degrees of freedom than observed variables. This is necessary feature of organised biological systems: the manifold hypothesis simply implies the presence of organisation.

Low-dimensional representations of biology capture the correlations and patterns in biological systems that result from interactions between components. Building mechanistic models into these representations can thus learn the mechanisms by which these correlations and patterns are generated. Basic implementations of this more mechanistic form of dimension reduction exist already for single-cell data. These include algorithms for describing the gene modules that capture the correlations between genes^132^, ‘meta-cells’ that capture the coarse-grained patterns of cell states^133^, and pseudo-time and trajectory analysis tools^134^ that can model the path of cellular differentiation that explain the observed patterns of cell types in a dataset.

Further to this, methods of ‘causal representation learning^45^’ aim to disentangle complex phenotypes into distinct biological processes and in doing so to learn causal relationships from the data^135–144^. These approaches have been applied to a wide range of biological contexts, with simulated and real single-cell genomics data, and offer the prospect of more explainable, generalisable models of complex biological systems that are grounded in theory of causal discovery.

These methods have thus far been applied largely to the problem of perturbation prediction: learning the causal effect of genetic and chemical interventions on cells. Further work applying these methods could provide mechanistic representation of how gene regulation systems drive cell and tissue level outcomes during development.

Such a model would look to abstract away molecular details, shifting the onus of these complexities to the abstract parameters of a neural network, freeing the meaningful parameters of the model to learn a smaller number latent causal factors that connect with or drive particular cellular phenotypes (Figure 4).

The challenge here is to constrain the model such that what is being learnt is meaningful for the question. For example, one could enforce latent variables to map to genes of a particular GO terms, or to particulars chromosomes, or that the mapping passes through a dynamical model of transcription. The constraints determine what the model learns. Examples of how GRN models could be constructed from additional forms of data and model structures are described in Box 2. These approaches provide models of gene regulation that are consistent with the observed genetic dynamics, while explaining higher level evolutionary or cellular phenomena.

If the evolution, cellular function or organismal application of GRNs dictates anything about their form or structure, building these details into our models can serve to reduce the vast solution space. Doing so in a representation learning framework can provide the ‘higher-level’ constraints that allow coarse-grained models to abstract the noise from the signal.

## Experimental solutions

Building a high-level understanding of gene regulatory function may not require mapping out every molecular component of a network. But just as the challenge of ‘dynamical equivalence’ means that many different model parametrisations can generate the same dynamics, many different molecular systems could generate equivalent mechanisms. Modelling gene regulation from measurements of molecular components in a way that is robust and generalisable across cell-types, tissues, organisms and species, requires understanding the molecular ‘rules’ that dictate gene regulatory function. The set of possible solutions for a gene regulatory model spans the space of possible molecular instantiations of the system, so to constrain the solution space for GRN models, we must understand what can and cannot happen at a molecular level.

**Figure 5:**
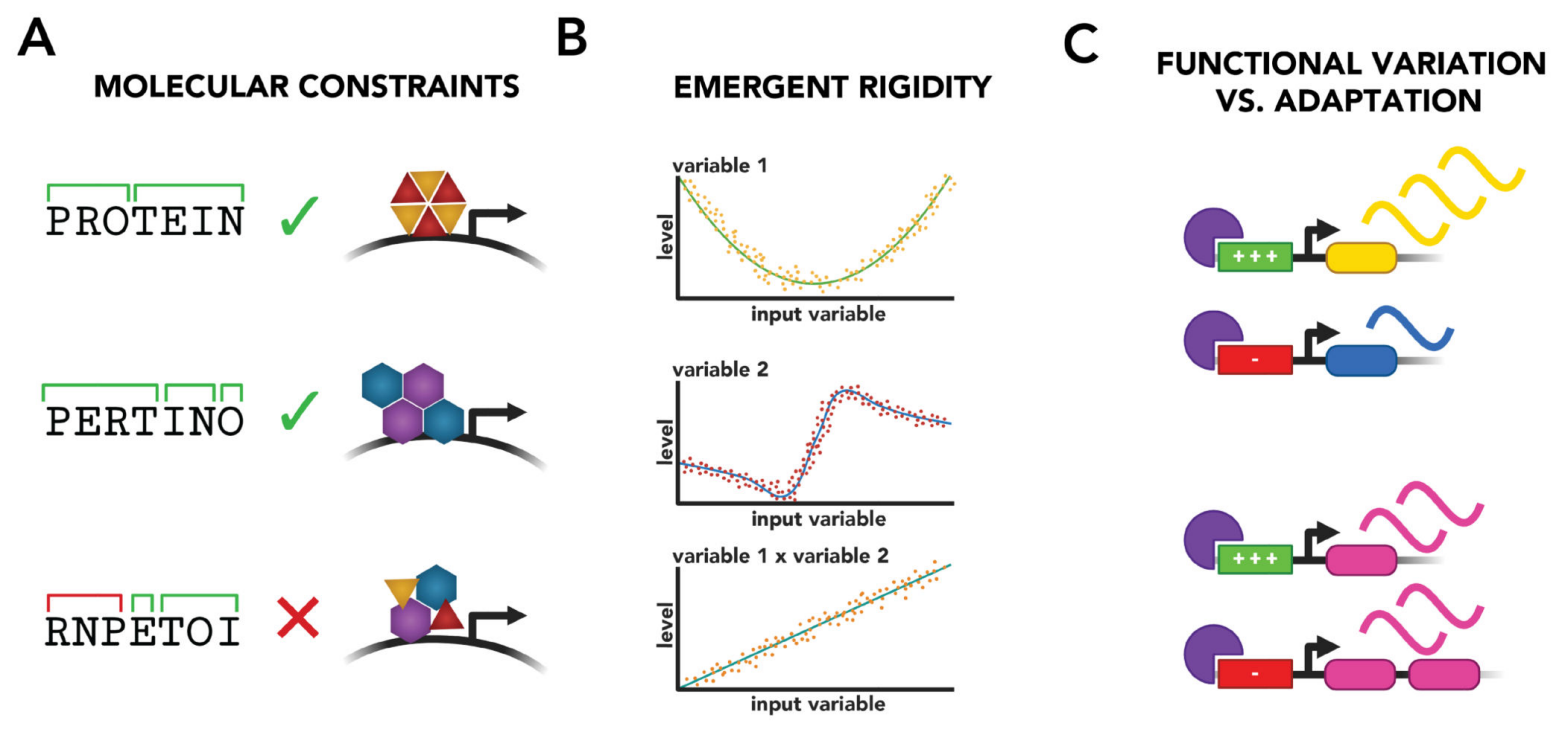
Establishing molecular principles governing GRNs. A. The structure of the English language can help constrain the solution space when determining whether a letter sequence is a valid word: both ‘protein’ and ‘pertino’ follow valid rules of phonetics, while the sequence ‘rnpetoi’ contains the invalid phonotactic cluster ‘rnp’, so can be ruled out. Similarly, understanding the structure of allowed configurations at the molecular level can help constrain the solution space for modelling GRNs (here demonstrated with the example of transcription factor complexes forming at the promoter of a gene). B. Relationships can appear from the interaction between seemingly non-predictive or flexible variables to create a form of ‘emergent rigidity’: here, neither variable 1 nor variable 2 show good correlation with the input variable, however the product of these two variables resolves into a clear linear relationship. C. Variation in biological mechanism (for example cis-regulatory enhancer activity) could be generated through two processes. Top: different enhancer activities/affinities can create dosage control between genes and between contexts, creating a functionally different output between contexts. Bottom: different enhancer activities/affinities can arise as an adaptation to an evolutionary event, such as the duplication of a gene. In this case, the variation does not create functional differences, but compensate for the previous evolutionary event.

As an example, consider the challenge of learning the ‘cis-regulatory code’ that maps the sequence of an enhancer to its function. An unbiased approach faces an intractably large solution space: there are more possible 200 base-pair sequences than atoms in the universe. Defining biological principles and design constraints reduces this space to that of biologically plausible mechanisms. Just as knowing linguistic structures, such as syllables and phonemes, helps identify valid words in a language, understanding molecular principles organising gene regulatory interactions provides a framework to link basic physical units (DNA bases and transcription factors) to their broader functional meaning (Figure 5A).

‘Big-data’ methods such as single-cell genomics will be vital for describing patterns of gene regulation across contexts. Yet understanding gene regulation requires moving beyond cataloguing and correlating these observations. It will be important to also capture how different regulatory layers connect.

Individual layers of regulation can give the impression of sloppy or noisy mechanisms: TF binding motifs are degenerate^61^, while enhancers are often redundant^145,146^. Transcription factors appear to bind thousands of sites in the genome, often in complexes of interchangeable membership, sometimes binding alongside other TFs that they also directly antagonise^147,148^. The role of other forms of regulation, such as histone modifications, DNA methylation and non-coding RNAs, also appear to display context-dependent function^149^. This leads to an impression of extreme, almost unlimited flexibility, from which remarkable robustness and precision emerge.

Robustness may be achieved through interactions between regulatory layers. Variations in one layer may be coupled to, complemented, or counteracted by variation in another to produce a form of ‘emergent rigidity’ (Figure 5B). For example, one set of observations might indicate that a gene is regulated by both distal and proximal enhancers, such that the genomic distance of these regulatory elements is not predictive of their relative activities. Another set of observations might find that this gene is contained within a 3D topology that is remodelled between different cell types. Integrating these observations might lead to the finding that the 3D organisation and genomic location of enhancers together resolve into a predictive model of cell-type specific enhancer activity^150^. Alternatively, one assay might document the sequence variability of motifs bound by a TF, while another might show that the TF induces the expression of targets that are not specific to a single cell type. Together, these findings could resolve into a model of dose-responsive TF binding, where motifs of different affinities drive the expression of different cell type programs (as has previously been shown to be the case for Sox2^151^). Examining only one layer of regulation may never provide mechanistic understanding: the cis-regulatory code^148,152^ of gene regulation may only exist in a vague sense when viewed solely from the lens of DNA sequence or TF activity; examining relationships between layers may allow a form of code to emerge more clearly.

For this, multimodal experimental designs linking perturbations in one layer to measurements of another will be valuable. Engineering sequence variation while measuring chromatin conformation with Hi-C; measuring chromatin accessibility changes in response to transcription factor overexpression^153–156^; recording enhancer activities across histone-code perturbations^157^ - experimentally linking regulatory processes may reveal how the many facets of gene regulation act in concert to constrain cell behaviour.

However, not all regulatory variation is necessarily functional. Variability can provide functional benefit (for example, binding motif degeneracy may allow the intensity of a TF’s effect to be modulated across contexts^151^) but could also occur through evolutionary chance. For example the duplication of a TF gene could lead to evolutionary changes in cis-regulatory sequences that accommodate two redundant regulators, rather than to removal of the duplicated gene (Figure 5C). Equally, variation can be driven by stochastic processes of mutation and recombination that do not generate strong enough selective forces to be eliminated through evolution^158^.

To test how structural aspects of a GRN maps to its function, we will need to re-arrange the structure of these networks ourselves.

By altering the composition and combination of cisregulatory elements in a cell, we can begin to alter the strength, polarity or presence of interactions between genes, providing an experimental exploration of how different network structures generate various phenotypes. Work in this direction is ongoing: high-throughput enhancer mutagenesis^159^, engineered rearrangement of the human genome^160^ and of enhancer landscapes^161^, high throughput enhancer knockout^162^ and TF induction screens^163^ exemplify this direction. In parallel, methods for designing cis-regulatory sequences towards specific regulatory functions are rapidly maturing, with enhancer screens capable of testing the cell-type specific regulatory activity of thousands of DNA sequences simultaneously^164,165^; detailed analyses of how structural recombination alters enhancer function^166^; saturation genome editing methods that measure functional readouts of exhaustively altered regulatory regions^167^; and machine learning methods for the *de novo* generation of cell-type and function specific enhancer sequences^168–170^.

These developments point towards a future of synthetic gene regulatory engineering, where cis-regulatory sequences and transcription factors can be edited or introduced to produce targeted alterations to the structure, and thus function, of gene regulatory networks. This, in turn, could create the possibility of designing entirely synthetic gene regulatory networks as objects of investigation, building on existing work in synthetic biology that has created programmable protein circuits^171^ and protein-level neural networks^172^ in mammalian cells, or ribocomputing devices^173^ in bacteria, or genetic logic circuits^174^ in yeast. Such efforts could provide gold-standard ground truth systems for building and benchmarking GRN modelling frameworks, but more broadly would open up a vast space of GRN structures beyond that of naturally occurring systems.

The experimental progression outlined above moves from outlining molecular rules and the connections between layers of regulation in a network, to re-arranging, re-designing and ultimately constructing new gene regulatory systems. This pathway offers the opportunity to build perturbation frameworks of commensurate sophistication to match the scale of our data-collecting abilities and the complexity of the systems we study. For theoreticians, it offers a particular enticing prospect: the dynamics of gene regulatory networks are, to a certain degree, written into the sequence of the genome. Editing gene regulatory networks through genome engineering thus offers a novel prospect for causal discovery, namely that we can systematically alter the structure of causal interactions in the system we wish to understand.

This opportunity is reminiscent of a recently proposed strategy for understanding cis-regulatory DNA code. This strategy suggested we should ‘hold out the genome:’^148^ the totality of naturally observed regulatory sequences is only a tiny proportion of the total possible sequence space, so to understand cis-regulatory sequences we must train models on larger libraries of synthetic sequences, expanding beyond what is observed in nature. A similar argument applies to gene regulatory networks: the total space of possible network structures is far larger than is observed in biological systems. By building new synthetic systems and re-engineering the structures of existing ones, we can generate a far deeper and more extensive exploration of gene regulatory networks than is currently possible.

## Perspective: Machine learning for biological networks

The rapid progress of biological technologies, from genomics techniques to computational methods, has created considerable excitement regarding our understanding of gene regulatory mechanisms and cellular function (see Box 4 for open challenges). This has led some to call for the construction of ‘virtual cells’^175,176^ and foundation models that provide ‘universal representations’ of cellular biology^7,177^.

One driving force for this excitement is the success of AlphaFold^92,178^, arguably the first *bona fide* biological foundation model. Creating a comparable model for gene regulatory systems requires one to shift scales from the molecular to the cellular. To appreciate the challenges associated with this shift, one must consider the differences between these contexts: crystal structure prediction is a static and clearly definable problem. It benefits from ground truths and robust metrics of success. Moreover, the Protein Data Bank (PDB) remains one of the cleanest and most consistent biological datasets ever constructed. Proteins display structural ‘degeneracy’, where structures contain commonly repeated motifs, such as alpha helices and beta sheets, making the challenge of structure prediction considerably more tractable.

By contrast, ‘cell function’ is a context-dependent concept with no objective definition, and ‘cell states’ can only ever be partially observed. Single-cell sequencing data are noisy, sparse and suffer from batch effects, meaning that collected databases require considerable levels of data processing and transformation to be integrated^179^. Unlike protein structure, cellular decision making is dynamic and context-dependent, such that seemingly identical cells can behave differently due to unobserved differences (for example clonal dynamics, variation in cell culture conditions, stochasticity of gene expression).

These differences in context highlight key areas where progress is required. For example, protein structure prediction models are not trained on raw data; X-ray diffraction densities are integrated, scaled and processed into atomic coordinates, which are then the input to these models. This process involves the incorporation of biological knowledge, specialised algorithms and human over-sight to produce a standardised representation. Moving genomics ‘harmonisation’ methods, which currently focus just on removing batch effects, towards producing refined and biologically- and biophysically-motivated ‘cell state’ data representations, analogous to atomic coordinate data, may be key to building foundation model-worthy datasets. Moreover, as genomics methods become cheaper and more widely available, we must shift focus from cells-per-experiment towards samples-per-experiment to capture denser sampling of timepoints, signalling contexts, perturbation conditions. When we can record thousands of samples per experiment, rather than thousands of cells, we may start to collect datasets that display the same degeneracy of repeated patterns that has proven so fruitful for protein structure prediction and evolutionary sequence modelling^180^.

The continued growth of multi-modal sequencing technologies could minimise the problem of cell states only ever being partially observed^41^, but these methods must provide robust, consistent datasets that can be continually re-used, as we have seen with the PDB. It is notable that the year in which the PDB began was the same year that Minsky and Papert published *Perceptrons*^181^, a pessimistic appraisal of the limited utility of neural nets as statistical models. In this year (1969), deep learning did not exist, the most sophisticated computers had only kilo-bytes of memory, and there was no sense that PDB would provide the data for a computational solution to protein structure. We must similarly plan to generate datasets with the size, scope and quality to be used for years to come with computational methods that do not yet exist.

Even as we construct robust datasets and clear modelling objectives to replicate the environment of AlphaFold, we must recognise the fundamental differences between protein structure prediction and GRN modelling. AlphaFold is a predictive method; the goal is not to learn the biophysical principles that govern protein folding, but to predict structure from amino acid sequence. The objective of a GRN model should not only be to predict the phenotypic consequence of a particular molecular genetic state, but also to learn the principles that connect these two scales.

A black-box model predicting cell phenotypes is insufficient. Interpretability is required. The challenge is that our conception of interpretability in biology exists largely at the molecular level: proteins and genes do things, they are the de facto mechanistic units in the cell^8^. Building an interpretable understanding at a systems-level will require new conceptual frameworks that define what a meaningful systems-level mechanism can look like.

Such frameworks will need to exploit the organisational features of GRNs, such as their hierarchy and modularity. Unlike protein folding, GRNs may possess a ‘representational’ layer that captures not just the molecular implementation, but the logic of how this implementation organises in response to inputs to deliver outputs. Parsing this representational logic has the potential to reveal new design principles, entirely new forms of mechanism, and new views of how information is controlled in biology.

These features of GRNs, hierarchy, modularity, DNA rules, regulatory layers, point us towards how GRN models could be learnt. Hierarchy and modularity imply that GRNs are reducible, allowing molecular complexity to be abstracted away. If rules exist in DNA sequence, then sequence editing can redesign the rules, allowing exploration of a vast space of re-engineered systems. The organisation that emerges from interactions between different regulatory layers may explain how robust cell fate decisions arise from seemingly noisy molecular processes. Evolutionary analysis can discriminate between historically contingent patterns and fundamental rules of regulatory logic.

Our current view of the GRN – a static graph of lines and nodes – provides limited insight into the cellular function of regulatory networks. This fact is in stark contrast to the original formulation of GRNs as causal molecular explanations^26^. But we are well positioned to return to a mechanistic view. Single-cell genomics can measure biological phenotypes at huge scale and high resolution. Machine learning can convert these data into simpler representations. By building cellular and evolutionary constraints into these representations, we can distil the complexity of gene regulatory systems into the core logic of developmental processes. Ensuring these models are meaningful will require experimental developments that set out the syntax and structure of molecular mechanisms, both in the principles of how they operate across contexts and the relationships that govern how different mechanisms interact. Synthetic biology methods that can build and manipulate gene regulatory systems will be transformative for this effort, providing a depth of understanding that would be impossible through examination of natural regulatory systems alone. Our goal in gathering more detailed datasets and building more sophisticated models should be to dismantle the complexity of gene regulatory networks and reveal the underlying design principles of developmental logic.

## Figures and Tables

**Figure 4:**
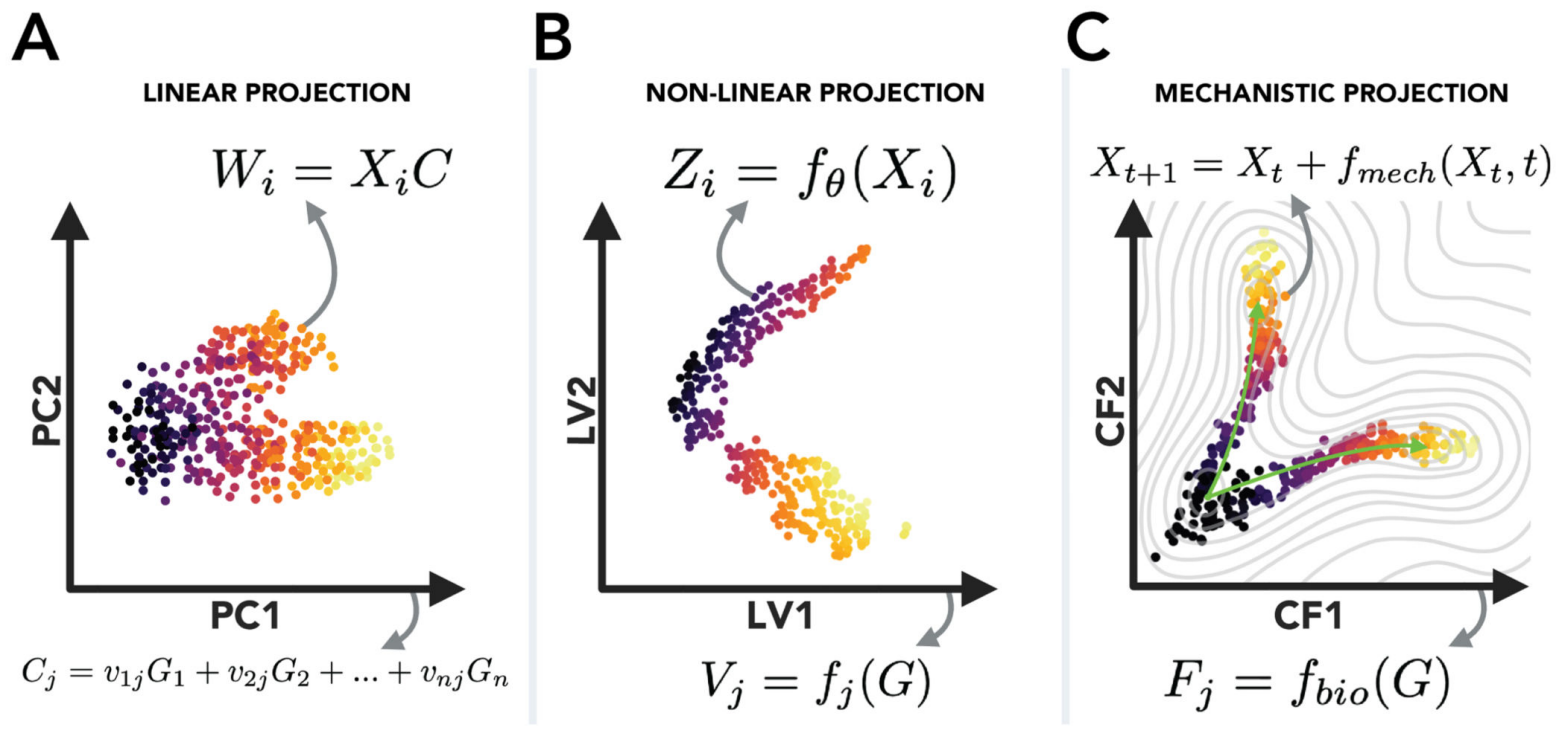
Towards mechanistic abstract representations. A. In principal component analysis (PCA), each projected data point is a linear transformation of the original data-point by a transformation matrix *C*. Accordingly, each component can be described as a linear combinations of variables in the original dataset (for example, genes in RNA sequencing data). B. Dimension reduction methods such as autoencoders, UMAP or t-SNE provide a generalisation of this idea beyond linear mappings, where each data point is mapped through a nonlinear function to a latent representation (to use the nomenclature of autoencoders). Hence, each latent variable can be described as a non-linear function of the variables (genes) in the original dataset. C. A mechanistic adaptation in which the latent representation is constructed from a mechanistic model (*f*_*mech*_) that captures the causal relationships between latent factors than can explain the dynamics of data-points (for example, sequenced cells). This mechanistic model could be a dynamical system describing the time-dependent progression of cells transitioning between cell states. In parallel, data variables (genes) are mapped to latent variables by a function that is subject to biological constraints (*f*_*bio*_), ensuring the mapping is biologically meaningful. The structure of the latent representation and the mapping from variables to latent factors are interdependent, but not equivalent (as with PCA). The mechanistic model can capture the causal relationships driving *cell level* behaviours, while the latent variable mapping learns how genes connect to these causal relationships.

## Box 1 Organisational Features of Gene Regulatory Networks (GRNs)

**Modularity:** GRNs can be decomposed into smaller sub-circuits, each responsible for a specific regulatory task. This modular organisation breaks the process of development down into manageable units.

**Conserved Sub-Circuits:** Similar sub-circuit topologies, although composed of different regulatory genes, perform comparable functions in diverse developmental contexts. Sub-circuits usually involve two or three regulators engaged in mutual feedback and ensure the stability of the regulatory state despite transient initial inputs. For instance, positive feedback sub-circuits, responsible for locking down regulatory states, are common.

**Hierarchical Structure:** GRNs display a hierarchical organisation, with multiple layers of regulatory interactions controlling developmental processes. This hierarchical structure reflects the sequential nature of development, with each layer building upon the regulatory states established by the preceding layers. At the final layer activation of effector genes are responsible for differentiation and cell biological features of the cells in a tissue. By contrast in upper layers combinatorial cross regulation plays a significant role in shaping the activity in lower layers.

**GRN Depth:** Deep GRNs involve numerous regulatory steps, and are often found in the initial formation of embryonic territories that require multiple layers of sub-circuits to achieve their developmental outcome. By contrast, shallow GRNs have fewer regulatory transactions between initial inputs and the activation of effector genes. The formation of a typical tissue involves the establishment of a regulatory state in a progenitor field, followed by subdivision into regulatory domains for subparts, while maintaining the overall regulatory state.

**Impact of Regulatory Changes on Evolution:** Extant GRNs are a product of evolutionary processes. The rewiring of GRNs appears to play a significant role in the evolution of novel structures. In addition to gradual modification, entire GRN circuits can be co-opted into new developmental contexts^49^, increasing GRN complexity and resulting in the generation of novel features. Modifications to the cis-regulatory elements of genes can modify the timing, location, or level of gene expression, impacting developmental processes.

## Box 2 Learning representations of GRNs

**Evolutionary dynamics:** by incorporating evolutionary data, and enforcing that each latent variable maps to genes with specific evolutionary dynamics, one could learn a representation of gene regulation that is consistent with the kernel theory of GRN evolution.

**Cellular signal processing:** by defining a GRN model as receiving only a cell’s signalling environment (for example in an in vitro system) as inputs and producing as output a cell fate, one could learn a representation of gene regulation that captures cellular signal interpretation.

**Proliferation vs. differentiation:** one could define latent variables that are parameters of an agent-based model that can simulate cellular decisions to either proliferate or differentiate (potentially using lineage tracing data).

**Sub-circuit modelling:** combining representational models with theory from systems biology could provide methods to treat latent variables as representing particular network motifs or common sub-circuit structures, producing a decomposed GRN model that captures the modular structure of regulatory networks.

## Box 3 Glossary

**Coarse graining:** a method commonly used in statistical mechanics and chemical simulation methods. Details at a lower scale of resolution (e.g. atomic level) are removed or averaged, such that only features that are essential to preserve macroscopic behaviour at a higher scale (e.g. bio-molecular) are retained. For example, only core geometrical features may be required for protein structure prediction, meaning the explicit atomic structures need not be considered.

**Ground truth:** the real-world model or outcome of a system, serving as a benchmark against which trained models can be compared to assess performance.

**Bayesian modelling:** a probabilistic modelling approach that combines prior beliefs (prior distributions) with observed data (likelihoods) to estimate an updated belief (posterior distributions), allowing inference and prediction with robust uncertainty quantification.

**Dynamical systems:** models of systems whose state evolves through time, often expressed in the form of differential equations. Often used to describe biological systems such as population dynamics, biochemical reactions and gene regulatory networks.

**Structural non-identifiability:** when wide variation in a model’s parameters produce small changes in model output, such that there is not a unique solution for a model’s fitting to a dataset. This issue is structural because it is not necessarily a consequence of poor data quality, but because of ambiguity or flexibility in the structure of a model; the larger a model becomes, the more probable that it will suffer from structural non-identifiability.

**Sloppy models:** related to structural non-identifiability, sloppy models are systems biology models that show extreme sensitivity to a small number of parameters, with the majority of parameters having no effect on model performance.

**Bijective mapping:** a function that creates a one-to-one correspondence between two sets, such that every element in one set maps to one and only one element in the other. If there existed a bijective mapping between the structure and behaviour of a GRN, knowing the structure would necessarily mean knowing the behaviour. Because interactions can have varying strengths and dynamics, the simple structure of interactions in a GRN does not have a one-to-one mapping to the GRN behaviour (a theoretical perfect parameterisation of a GRN model might, but issues of structural non-identifiability may even prevent this from being true).

**Multi-scale:** models or analysis that represent phenomena across different scales of resolution in time, space or organisation, for example the atomic, molecular, bio-molecular, genetic, cellular, organismal and population scales of biology.

**Multi-perspectival:** the idea that the representation of a phenomenon may depend on the viewer’s perspective or researcher’s question. The most useful representation of a gene regulatory system may differ for example for questions concerning global cell type control versus questions concerning regulation of specific genes.

**Marr’s levels of analysis:** a framework introduced by David Marr to describe cognitive and computational systems that breaks these systems into three layers: the computational level, which provides the broadest description of what the system does and why; the algorithmic/representational level, which captures the steps and organisation of the system required to fulfil the computational purpose; and the implementational level, which describes the physical material and substrates used to construct the algorithmic organisation of the system.

**Dimension reduction:** a class of methods used to reduce the number of variables in a dataset while retaining the major sources of variation in the data. Methods such as PCA, UMAP and t-SNE are commonly used to reduce the dimension of sequencing data, either for visualisation or to create representations that are less noisy or sparse, and thus more tractable for downstream analysis.

**Representation learning:** related to dimension reduction, a field of machine learning focused on learning meaningful, compact representations of data, rather than using only the variables observed in the original data. The variables in these representations are considered ‘latent’ factors because they are not directly observed. Examples of representation learning models include the variational autoencoder (VAE), a type of deep generative model that encodes data to a latent representation, before decoding this representation back to reconstruct the original data. Like PCA, UMAP and other dimension reduction approaches, when the latent representation of a VAE is lower dimension than the data, this approach can be used to ‘bottleneck’ information, retaining only the most useful variation required to construct the original dataset. Unlike PCA or UMAP, VAEs can easily be modified with additional constraints, allowing biological or dynamical information to be incorporated into the latent features that are learnt.

## Box 4 Open Challenges

**Computational:**

- Creating new levels of abstraction for GRN models and linking between levels
- Developing causal representation learning methods
- Integrating cellular and evolutionary information into models
- Extending understanding of common GRN motifs to generalised cases
- Developing models that explicitly handle the dynamic nature of GRNs

**Experimental:**

- Creating reliable ground truth datasets from natural and synthetic systems
- Identifying molecular principles giving rise to GRNs across contexts
- Developing methods to understand CREs and gene regulation (single-locus proteomic assays, dynamic spatial assays, 3D genome)
- TF binding dynamics at single-molecule resolution
- Developing cis-regulatory manipulations and more sophisticated perturbations

**Technical:**

- Improving single-cell methods to capture more conditions per experiment
- Getting better direct information on TF–enhancer interactions
- Making multi-modal measurements more affordable and consistent
- Developing methods to track dynamic changes in GRNs

**Conceptual:**

- Understanding how different regulatory layers interact
- Bridging the gap between molecular interactions and cellular behaviours
- Determining appropriate levels of abstraction for different questions
- Understanding how evolutionary constraints shape network architecture
